# The Impact of Split Radiation Therapy on the Management of Locally Advanced Cervical Cancer in Central Virginia

**DOI:** 10.7759/cureus.83130

**Published:** 2025-04-28

**Authors:** Nophar T Yarden, Catherine Sport, Claudia Bale, Nitai Mukhopadhyay, Emma C Fields

**Affiliations:** 1 Radiation Oncology, Virginia Commonwealth University School of Medicine, Richmond, USA; 2 Gynecologic Oncology, Virginia Commonwealth University School of Medicine, Richmond, USA; 3 Biostatistics, Virginia Commonwealth University School of Public Health, Richmond, USA; 4 Radiation Oncology, Virginia Commonwealth University, Richmond, USA

**Keywords:** high-dose rate (hdr) brachytherapy, locally advanced cervical cancer, pelvic external beam radiotherapy, radiotherapy, treatment duration

## Abstract

Background and objective

Over the past few years, the complexity of brachytherapy (BT) has increased, and the practice patterns have shifted to distinguish high-volume centers as primary sites for these procedures. As a result, women with locally advanced cervical cancer (LACC) who are treated with external-beam radiotherapy (EBRT) at local centers are now more likely to be referred to higher-volume centers for their final BT boost. The impact of splitting radiotherapy sites on treatment adherence and outcomes is unclear. The purpose of this study was to compare the duration of treatment, recurrence, and survival between patients who received all radiotherapy at one center compared to those with split treatment.

Methods

A retrospective chart review was completed to identify women with stage IB-IVA cervical cancer treated with definitive radiation therapy (RT), including EBRT and BT between 2018 and 2023. Patients were grouped by location of EBRT, either at the primary institution (PI) or at an outside center. Patients were excluded if they had incomplete radiation therapy data, a missing address/zip code, metastatic disease, or a prior hysterectomy. Variables collected included demographics (age, race, ethnicity, insurance status, or geographic setting), disease and treatment characteristics, comorbidities, distance traveled to the RT sites, treatment duration, and survival status. Recurrence and survival analyses are limited to patients with at least one year of follow-up.

Results

Of the 66 women included in this study, 24 (36.3%) underwent EBRT at an outside location and were included in the split RT group. There was no significant difference between the two groups regarding age, disease characteristics, or comorbidities. The mean distance traveled to the PI was compared between the two groups and found to be statistically significant (p=0.001, t-test), with patients in the split group traveling a mean of 66.7 miles compared to 39.1 in the PI-only group. Likewise, the distance traveled to the EBRT site was significant, with women in the split group traveling a mean of only 13.6 miles compared to 39.1 (p<0.001, t-test). Of the 42 patients treated exclusively at the PI, 95.2% completed treatment within the recommended 56 days as opposed to 54.2% of the split RT patients (p<0.001, chi-squared test). Additionally, overall survival data were not significant; 80.8% of women in the PI-only group are reported to be alive without disease compared to 90.0% in the split group (p=1.000, chi-squared test).

Conclusions

In this study, we observed similar outcomes between LACC patients who had split their RT and those who received both EBRT and BT at the same high-volume PI. Yet, women who received RT at the PI exclusively had a shorter median duration of treatment and were more likely to complete treatment within the recommended timeline. Given the known relationship between treatment duration and patient outcomes in LACC, this study highlights the need to address factors that protract treatment duration to reduce potential disparities in care.

## Introduction

The standard of care for locally advanced cervical cancer (LACC), defined as stage IB3-IVA, includes external-beam radiation therapy (EBRT) with concurrent chemotherapy followed by brachytherapy (BT) [1,2]. The duration of this treatment regimen has been studied and strongly linked to patient outcomes. In the 2024 NCCN cervical cancer guidelines, optimal results are achieved when the course is completed within eight weeks [1]. Specifically, studies have found that from the initiation of EBRT, overall treatment time longer than 56 days has been associated with lower rates of pelvic control [3,4,5]. Of note, the retroEMBRACE study found that completion of radiotherapy within 50 days provided a higher three-year local control rate than patients treated over >50 days [6]. For patients who travel a long distance to a radiation center or have other barriers to care, this time constraint adds logistical challenges in receiving the treatment. 

With the development of image-guided adaptive brachytherapy, the use of volumetric imaging, and hybrid applicators, the complexity and required resources for treating cervical cancer with brachytherapy have increased. Over the last several years, there has been a noticeable decline in providers offering brachytherapy in our outreach areas; as a result, most patients are now referred for brachytherapy at our center after completion of their EBRT [7]. [1] Our center draws patients from a large geographic area with vast socioeconomic gaps [8]. These patients may be at increased risk for prolonged treatment time, given that they are traveling longer distances for BT treatment, and coordination of care is shared between multiple sites. Previous reports have identified the distance traveled to radiation therapy (RT) centers as a modifiable barrier to healthcare access. Yet, recent studies have suggested that the role of distance to RT centers in predicting cervical cancer outcomes is complex [9]. 

Interestingly, a study from the University of Virginia evaluated the impact of distance to the radiation facility and found no significant effect on progression-free and overall survival; however, this study identified that federal insurance or lack of insurance coverage were the most important factors in predicting risk of death [10]. A similar single-institution study found that compared to women who received all of their treatment at one center, those who underwent EBRT and BT at different facilities were more likely to have protracted courses of treatment, which in turn led to poorer overall survival [11].

This study aims to compare the distance to the treatment site, treatment duration, and outcomes in patients receiving EBRT and brachytherapy at different locations versus patients receiving all radiation treatments in the same facility. We hypothesize that patients receiving all care at one center will have improved adherence and thus shorter time to treatment completion. The primary outcome of this study is time to completion of the entire course of RT.

Preliminary results relating to this article were presented as abstracts at the American College of Radiation Oncology Summit on March 16, 2023.

## Materials and methods

Following Institutional Review Board approval, we performed a retrospective review of women with stage IB3-IVA cervical cancer treated between 2018-2023. Women aged 18-100 years old treated with definitive intent RT and who received BT at the primary institution were included. Patients were identified using the Aria Radiation Oncology and Epic SlicerDicer databases. Patients were excluded if they had incomplete RT data, missing address and zip code, metastatic disease, or prior hysterectomy.

Electronic medical records were reviewed to collect data on patient demographics, cancer variables, and treatment course, including age, race, ethnicity, geographic setting, insurance, medical comorbidities (hypertension, diabetes, tobacco use, and BMI), disease characteristics, treatment details, and outcomes. 

To calculate the distance each patient traveled for treatment, the distance from the patient’s home address to the EBRT center (either the primary institution or outside center) and the primary institution was calculated using Google Maps. The home address was identified using the address listed on the electronic medical record at the time of data collection. If no address was listed, the patient’s zip code was used. The geographic setting was determined using the United States Department of Agriculture’s (USDA) Rural-Urban continuum codes based on the 2013 dataset, which was last updated on December 10, 2020 [12]. Using county codes, patients’ addresses were classified as metropolitan, micropolitan, small town, or rural. The population cutoffs for geographical settings were based on the following cut-offs: metropolitan ≥50,000 residents, micropolitan: 10,000 - 49,999, small town: 2,500 - 9,999, and rural <2,500. Survival and recurrence data were obtained by reviewing updated medical records, physician notes from outside centers, and online searches for relevant obituaries, as needed. Recurrence was determined based on chart review and calculated from the end date of RT to the date of the last clinical follow-up. Survival was calculated from the end date of RT to the date of death. 

The patients that met inclusion criteria were then divided into two data groups for analysis by treatment site; The “ primary institution (PI)-only group” were those who had all of their RT (EBRT and BT) at the primary institution (n=42) and the “split group” were those who had EBRT at an outside institution with only BT at the primary institution (n=24). 

The study’s primary endpoint was the time to completion of RT, defined as the time (in days) from the first radiation treatment to the last BT treatment. The time to completion of radiation treatment was evaluated as both a continuous and categorical (≤56 and >56 as well as ≤50 and >50) value. Univariate analysis was done using t-tests for continuous variables and chi-squared tests for categorical variables. Multivariable analysis was completed for demographic variables with predicted significant differences between the two groups using the Cox proportional hazard model. A p-value <0.05 was considered statistically significant. Statistical analyses were performed using the statistical software R v 4.2.1.

## Results

A total of 66 patients met all inclusion criteria; the average age of the cohort was 52 years (range: 22 - 82 years), with no significant difference between the groups (Table 1). The racial and ethnic composition of the two groups was not statistically significant (p=0.848, p=0.288, respectively, for the chi-squared test). Differences in insurance status rates were not significant, although a higher percentage of patients in the PI-only group were uninsured (14.3%, six patients) compared to none in the split group (chi-squared p=0.145). There were no notable differences in patient comorbidity status related to diabetes, hypertension, tobacco use, or BMI. Differences in the geographical settings of the patients’ addresses were found to be statistically significant, with split patients more likely to reside in metropolitan counties (83.3%, 20 patients), whereas PI patients were more likely to live in micropolitan and small-town settings (chi-squared p=0.006).

**Table 1:** Demographics ^a^Of the patients listed under Other, three are Asian (one in outside, two in PI), and 11 preferred not to report or had missing data (five outside, s PI). ^b^Area of residence is defined using the patient’s county FIPS number using the United States Department of Agriculture’s Rural-Urban continuum codes based on the 2013 dataset, which was last updated on 12/10/2020. The following cutoffs were used to define the geographical setting: metropolitan: 50,000 residents, micropolitan: 10,000-49,999 residents, small town: 2,500-9,999 residents, and rural: <2500 residents. ^c^Distance is reported in miles using Google Maps. Distance was calculated from the patient's address to the clinic address. ^*^Denotes p-value from t-test. ^@^Denotes p-value from the chi-squared test EBRT: external-beam radiotherapy; PI: primary institution; SD: standard deviation

Demographics	Split (n=24)	PI (n=42)	P-value
Age at diagnosis, years, mean (±SD)	53.7 (±12.8)	49.6 (±14.6)	0.239^*^
Race, n (%)			0.848^@^
Caucasian	12 (50.0%)	23 (54.8%)	
Black	6 (25.0%)	11 (26.2%)	
Other^a^	6 (25.0%)	8 (19.0%)	
Ethnicity, n (%)			0.288^@^
Not Hispanic/Latino	24 (100%)	38 (90.5%)	
Hispanic/Latino	0	4 (9.52%)	
Insurance status, n (%)			0.145^@^
Private	7 (29.2%)	12 (28.6%)	
Federal	17 (70.8%)	24 (57.1%)	
None	0	6 (14.3%)	
Area of residence^b^,n (%)			0.006^@^
Metropolitan	20 (83.3%)	19 (45.2%)	
Micropolitan	2 (8.33%)	17 (40.5%)	
Small town	1 (4.17%)	5 (11.9%)	
Rural	1 (4.17%)	1 (2.38%)	
Distance traveled to PI^c^, miles, mean (±SD)	66.7 (±30.6)	39.1 (±31.7)	0.001^*^
Distance traveled to EBRT facility, mean (±SD)	13.6 (±9.2)	39.1 (±31.7)	<0.001^*^

The tumor stage and characteristics were similar between the two groups (Table 2). Approximately 82.0% of women (54 patients) had squamous cell carcinoma, 11 (17.0%) had adenocarcinoma, and one had small cell carcinoma. Ten (15.2%) had International Federation of Gynecology and Obstetrics (FIGO) stage I, 22 (33.3%) FIGO stage II, 31 (47.0%) FIGO stage III, and three (4.5%) FIGO stage IV. The associated letters for FIGO staging are presented in Table 2, though the represented p-value is shown for the numbered FIGO stage only. Fifty-eight women (83.7%) received platinum-based chemotherapy in addition to RT. 

**Table 2:** Disease characteristics ^@^Denotes p-value from the chi-squared test FIGO: International Federation of Gynecology and Obstetrics; PI: primary institution

Disease characteristics, n (%)	Split (n=24)	PI (n=42)	P-value
Histology			1.000^@^
Squamous	20 (83.3%)	34 (81.0%)	
Adenocarcinoma	4 (6.7%)	7 (16.7%)	
Small cell carcinoma	0	1 (2.4%)	
FIGO stage			0.356^@^
IB	4 (16.7%)	6 (14.3%)	
II	8 (33.3%)	14 (33.3%)	
IIA	2 (8.3%)	4 (9.5%)	
IIB	6 (25.0%)	10 (23.8%)	
III	12 (50.0%)	19 (45.2%)	
IIIB	5 (20.8%)	2 (4.8%)	
IIIC	7 (29.2%)	17 (40.5%)	
IVA	0	3 (7.14%)	
Chemotherapy			0.624^@^
Cisplatin	21 (87.5%)	37 (88.1%)	
Other	1 (4.17%)	0	
None	2 (8.3%)	5 (11.9%)	

The median distance traveled from the patients’ address to the PI for all patients was 51.1 miles (range 1-133 miles). The mean overall distance traveled to the PI was compared between groups and found to be statistically significant, with split group patients traveling a mean of 66.7 miles (range 5.1-133 miles) and PI-only patients traveling an average of 39.1 miles (range 1-127 miles) (t-test p=0.001). Additionally, the mean distance traveled to the EBRT site was found to be statistically significant between the two groups, with women in the split group traveling to outside sites a mean of only 13.6 miles (range 1.9-29.6 miles) (t-test p<0.001). 

**Figure 1:**
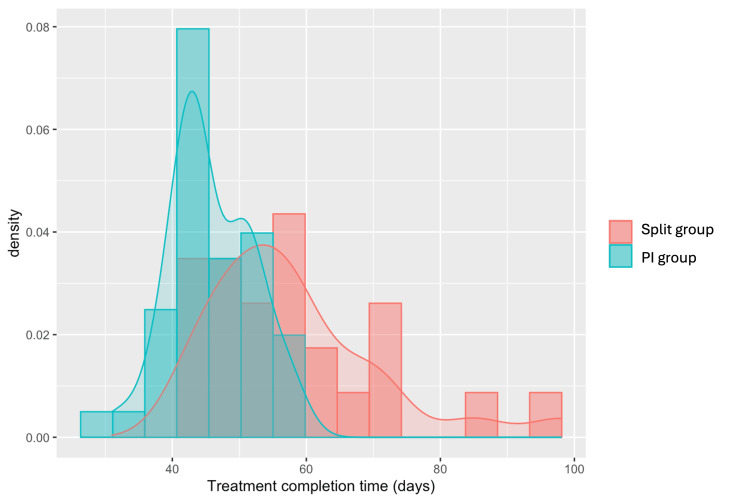
Treatment duration PI: primary institution

The median time to treatment completion in the PI group was 44.0 days (IQR: 8.8 days) compared to 56.0 days (IQR: 13.0 days) in the split group (t-test p<0.001) (Table 3, Figure 1). Of the 42 patients treated at the PI only, 30 (71.4%) completed treatment within 50 days compared to only eight (33.3%) in the split group (chi-squared p=0.006); Likewise, 95.2% of the PI group completed treatment within the recommended 56 days in the PI group compared to 45.8% in the split group (chi-squared p<0.001). 

**Table 3:** Treatment duration ^*^Denotes p-value from t-test. ^@^Denotes p-value from the chi-squared test PI: primary institution

Treatment duration	Split (n=24)	PI (n=42)	P-value
Time to treatment completion			
Median (25^th^, 75^th^ quartile)	56.0 (49.8, 62.8)	44.0 (42.2, 51.0)	<0.001^*^
Treatment duration ≤50 days, n (%)	8 (33.3%)	30 (71.4%)	0.006^@^
Treatment >50 days	16 (66.7%)	12 (28.6%)	
Treatment duration ≤56 days, n (%)	13 (54.2%)	40 (95.2%)	<0.001^@^
Treatment >56 days	11 (45.8%)	2 (4.8%)	

**Figure 2:**
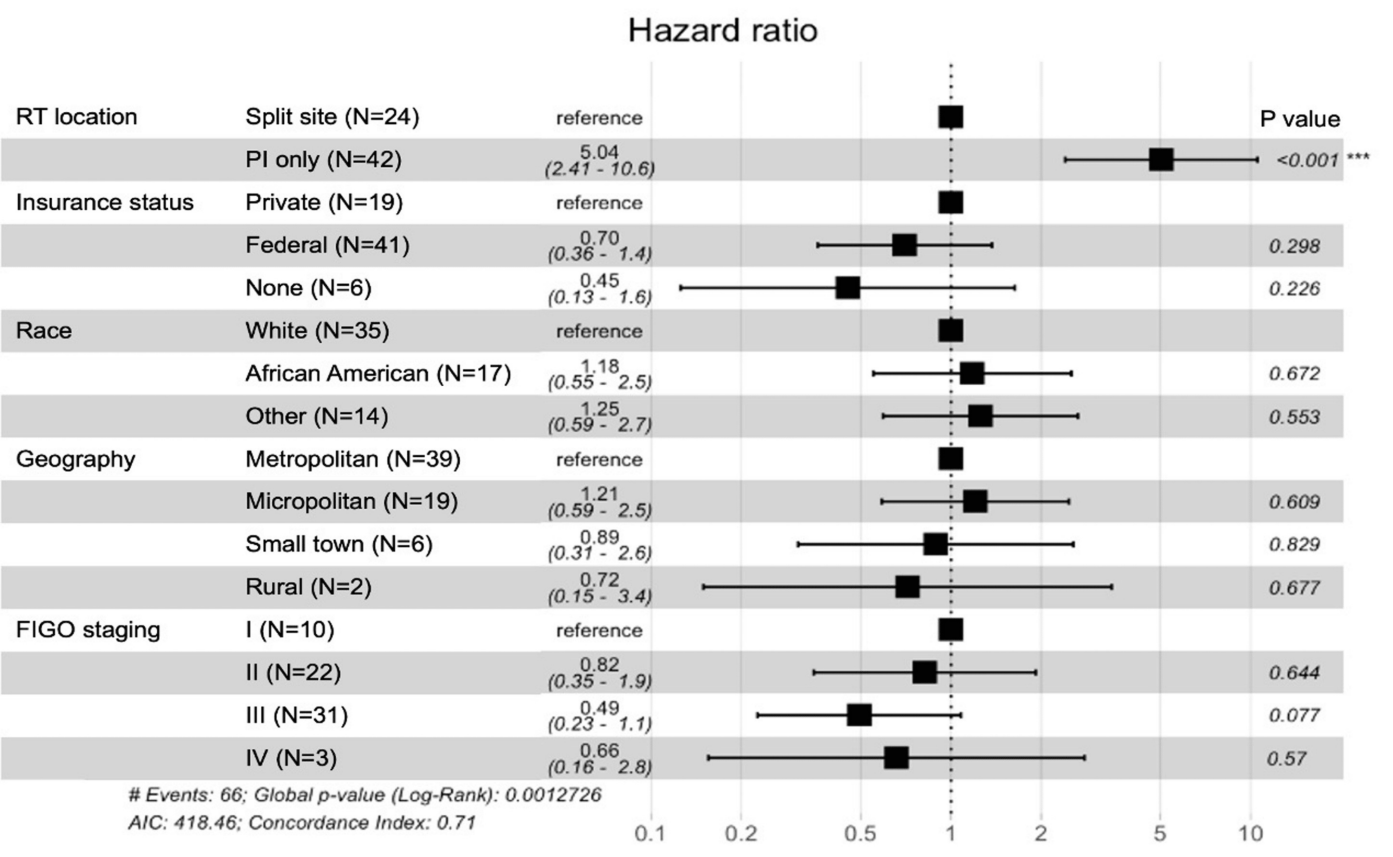
Multivariate analysis of treatment duration P-value is representative of the Wald test analysis. P-value <0.05 was considered statistically significant FIGO: International Federation of Gynecology and Obstetrics; PI: primary institution; RT: radiotherapy

On multivariate analysis, split treatment was the most significant factor associated with extended treatment time (Wald test p<0.001). Patients who had split treatment between two centers were significantly more likely to have a treatment duration greater than 56 days, as noted by a hazard ratio of 5.04. No significant associations were noted for treatment duration in evaluating the role of geographical setting, insurance status, race, or FIGO stage (Figure 2, Table 4).

**Table 4:** Multivariate analysis of treatment duration EBRT: external-beam radiotherapy; FIGO: International Federation of Gynecology and Obstetrics; PI: primary institution

Variables	Median treatment duration (days)	P-value (Wald test)
EBRT location outside of PI	56	
EBRT location at PI	44	<0.001
Private insurance	46	
Federal insurance	50	0.298
No insurance	47.5	0.226
Race: Caucasian	49	
African American	49	0.672
Other	48	
Geographical setting: metropolitan	51	
Micropolitan	44	0.609
Small town	44.5	0.829
Rural	51.5	0.677
FIGO stage I	45	
II	49	0.644
III	51	0.077
IV	39	0.57

Preliminary results of recurrence and survival data are presented in Table 5. The analysis is restricted to patients with at least one year of follow-up [n=36: PI (n=26) and split (n=10)]. No statistically significant differences were found in recurrence rates, overall survival, and patient status. The median follow-up was seven months for patients in the split-treatment group and 20 months for those treated at the primary institution only. Overall median follow-up for the entire cohort was 17.5 months.

**Table 5:** Summary of recurrence and death (patients with ≥1 year of follow-up) PI: primary institution

Patient outcome	Split (n=24)	PI (n=42)	P-value (chi-squared)
Recurrence, n (%)			1.000
Yes	1 (10.0%)	3 (11.5%)	
No recurrence	9 (90.0%)	23 (88.5%)	
Mean time from radiation end to recurrence, months	7.0	11.5 (±4.9)	
Status, n (%)			1.000
Alive without disease	9 (90.0%)	21 (80.8%)	
Alive with disease	1 (10.0%)	4 (15.4%)	
Died from disease	0	1 (3.8%)	

## Discussion

Given the known relationship between treatment duration and patient outcomes in LACC, this study highlights the need to address factors that protract treatment duration to reduce potential disparities in care. The results of our study indicated that women who received their complete course of RT at one facility had a 12-day shorter median treatment duration when compared to the split group. Similarly, the rates of achieving treatment completion within both the 50 and 56-day benchmarks were significantly higher in the PI group. Modifiers of treatment duration may be attributed to patient-related factors (e.g., disease stage and socioeconomic variables) as well as treatment-related factors (e.g., facility resources and coordination of care).

The role of socioeconomic factors, including insurance status, race, and distance from treatment centers, has been shown as a barrier to care, especially in patients with cervical cancer [13-17]. Two recent studies showed that Medicaid and uninsured patients are less likely to have timely initiation of treatment and more likely to have prolonged EBRT compared to privately insured patients [14]. Interestingly, our study showed no significant differences in insurance status, although a higher percentage of patients in the split group had federal insurance (70.8%) compared to the PI group (57.1%). However, no patients in the split group were uninsured compared to 14.3% in the PI group. A similar single-institutional study in 2018 explored racial disparities related to time to completion of EBRT and BT in cervical cancer treated with definitive chemoradiation [16]. Their results identified that non-white women were more likely to have prolonged treatment duration, yet this difference was not observed when adjusting for insurance status. Overall, their study found that patients with public insurance had more treatment delays, regardless of race [16]. In our study, racial and ethnic composition was even between the groups and not found to be a significant modifier of treatment duration in multivariate analysis. 

Distance traveled to care and geographic settings have been associated with protracted treatment duration. Previous studies have shown that rural residents are 78% more likely to have prolonged therapy compared to their urban counterparts [17]. Interestingly, within the same geographical setting, longer distances to care have been associated with prolonged treatment duration [9]. Women with stage IB2-IVA cervical cancer residing in rural areas and further from care were more likely to complete treatment on time compared to rural patients closer to care facilities. However, distance was not associated with treatment duration in urban patients. Overall, Spees et al. concluded that distance may present a greater barrier to access for rural patients [9]. In our population, most of the patients in the split group resided in metropolitan areas (83.3%), whereas in the PI-only group, most patients were either in metropolitan (45.2%) or micropolitan (40.2%) areas. The limited number of patients in small-town and rural areas, as noted in Table 1, may explain why the geographic setting was not identified as a significant variable for treatment duration in our multivariate analysis. 

It has also been proposed that differences in treatment duration between urban and rural patients may be related to volume at the patients’ respective care facilities [17]. Some studies have proposed that higher facility volume has been found to correlate with shorter treatment durations, as well as improved survival and adherence to standard therapy [18]. The Society of Gynecologic Oncology and the American Brachytherapy Society recommend receiving care at high-volume centers as these are associated with higher-quality care [19].

Furthermore, over the past few years, the complexity of BT has increased, and the practice patterns have changed to distinguish high-volume centers, particularly centers recognized by the National Cancer Institute (NCI), for these specialized procedures [20]. Unfortunately, access to such NCI-designated centers is fairly limited; only 64 cancer centers across the United States are currently recognized by NCI, and 14 states have none [20]. As a result, there are limited locations for women to receive comprehensive care for LACC, and it has become more common for women to receive EBRT at one location and then come to a larger center for BT [19]. A significant modifiable factor in reducing the time to completion of treatment is the coordination amongst physicians and healthcare systems [9]. Larger care centers are often more resource-intensive environments that allow for more organizational infrastructure and care coordination [13]. Unfortunately, patients transferring care between centers can have delays exacerbated by the resource limitations of tertiary and outreach centers. 

Overall, our study showed no differences in recurrence or survival outcomes between the two groups, however, we had a limited number of patients with sufficient follow-up. Currently, there are conflicting results related to the location of cervical cancer RT and differences in outcomes. Calo et al. found that a larger percentage of patients who had EBRT at an outside facility were more likely to report a treatment duration greater than 60 days (t-test p=0.005) and that these women had a higher rate of recurrence compared to those patients who received all RT at the PI [10]. In contrast, a study in Virginia did not find statistically significant differences in treatment duration, progression-free survival, or overall survival between patients treated at one site for BT or those with split treatment. Although their results trended toward patients with split RT having higher rates of recurrence [9]. As a result, the role of radiotherapy treatment location in patient outcomes continues to be an area of interest. 

In 2022, a retrospective study using the US National Cancer Database found that only 29.3% of Americans with LACC from 2004 to 2015 received chemoradiation and BT within eight weeks. This study proposed that poor access to BT is a primary limitation to standard-of-care treatment that disproportionately impacts women of Black race, non-private insurance, lower income, and rural residents [21]. This was also highlighted in a 2019 retrospective study that compared factors impacting overall treatment duration; this found that the largest culprit was the inability to initiate and/or complete cervical BT within the recommended window. This study notably reported that BT within an integrated care network was associated with shorter treatment duration [13]. It is therefore postulated that a strong focus on expanding access to BT may improve outcomes and reduce the disparities discussed in this paper. Continued efforts should be made by encouraging physicians and ancillary staff to coordinate BT early following patient diagnosis, addressing patient-related difficulties, such as travel and distance, and ensuring that radiation oncology residents have training in cervical cancer BT. 

Limitations

Our study is limited by a small sample size at a single institution that may not be generalizable to a broader population. Patients are limited to those treated from 2018 to 2023 to reduce confounding factors related to advancement in both image-guided RT as well as BT delivery. Although our population is limited, the patient demographics and comorbidities were balanced between the groups. Additional demographic factors could be assessed, including employment status, access to transportation, family support, and marital status, as well as history of substance abuse and incarceration. Furthermore, limited follow-up of patients may be an additional limitation of this study. Future studies should target multiple centers with a larger sample size to generate more generalizable results with longer follow-up.

## Conclusions

Patients with LACC living far from large or NCI-recognized centers, who elect to receive EBRT at a local center and travel for their BT fractions, are at increased risk of prolonged treatment duration. In part, this may be attributed to the increased requirement for coordination of care as well as the burden of additional travel on patients. In this analysis of women treated for LACC in Central Virginia between 2018 and 2023, patients who had split their treatment sites for EBRT and BT traveled significantly further for treatment of BT and less for EBRT. Overall median treatment duration in patients who received all RT at one site was notably shorter compared to those who had split treatment between two sites. No differences were noted in disease recurrence or survival between groups, though data were limited to 36 patients with at least one year of follow-up. Based on the known correlation between LACC RT within 56 days and disease control, further steps should be taken to address disparities in care coordination.

## References

[1] (2025). National Comprehensive Cancer Network. Cervical cancer. http://www.nccn.org/professionals/physician_gls/pdf/cervical.pdf.

[2] Han K, Milosevic M, Fyles A, Pintilie M, Viswanathan AN (2013). Trends in the utilization of brachytherapy in cervical cancer in the United States. Int J Radiat Oncol Biol Phys.

[3] Vitzthum L, Yuan J, Jones D, Boldt A, Dusenbery K (2019). Reducing prolonged chemoradiation treatment times for cervical cancer. BMJ Open Qual.

[4] Lin SM, Ku HY, Chang TC, Liu TW, Hong JH (2017). The prognostic impact of overall treatment time on disease outcome in uterine cervical cancer patients treated primarily with concomitant chemoradiotherapy: a nationwide Taiwanese cohort study. Oncotarget.

[5] Song S, Rudra S, Hasselle MD (2013). The effect of treatment time in locally advanced cervical cancer in the era of concurrent chemoradiotherapy. Cancer.

[6] Tanderup K, Fokdal LU, Sturdza A (2016). Effect of tumor dose, volume and overall treatment time on local control after radiochemotherapy including MRI guided brachytherapy of locally advanced cervical cancer. Radiother Oncol.

[7] Gill BS, Lin JF, Krivak TC (2014). National Cancer Data Base analysis of radiation therapy consolidation modality for cervical cancer: the impact of new technological advancements. Int J Radiat Oncol Biol Phys.

[8] Morris BB, Hughes R, Fields EC, Sabo RT, Weaver KE, Fuemmeler BF (2023). Sociodemographic and clinical factors associated with radiation treatment nonadherence and survival among rural and nonrural patients with cancer. Int J Radiat Oncol Biol Phys.

[9] Spees LP, Wheeler SB, Varia M (2019). Evaluating the urban-rural paradox: the complicated relationship between distance and the receipt of guideline-concordant care among cervical cancer patients. Gynecol Oncol.

[10] Calo C, Elliott JO, Clements A, Reid G, Rath K (2019). Cervical cancer radiation therapy compliance rates based on location of radiation therapy. Gynecol Oncol.

[11] Rauh LA, Saks EJ, Nakad-Rodriguez D, Showalter TN, Duska LR (2018). Cervical cancer care in rural Virginia: the impact of distance from an academic medical center on outcomes & the role of non-specialized radiation centers. Gynecol Oncol.

[12] (2025). United States Department of Agriculture. “Rural-Urban Continuum Codes.” USDA ERS - Rural-Urban Continuum Codes. USDA, 10 Dec.

[13] Valakh V, Coopey BC (2019). Factors associated with duration of overall treatment time for cervical cancer treated with definitive chemoradiotherapy. Cureus.

[14] Churilla T, Egleston B, Dong Y (2016). Disparities in the management and outcome of cervical cancer in the United States according to health insurance status. Gynecol Oncol.

[15] Wu J, Huang Y, Tergas AI (2020). The effect of guideline-concordant care in mitigating insurance status disparities in cervical cancer. Gynecol Oncol.

[16] Petersen SS, Doe S, Buekers T (2018). Definitive radiation therapy for cervical cancer: non-white race and public insurance are risk factors for delayed completion, a pilot study. Gynecol Oncol Rep.

[17] Tergas AI, Neugut AI, Chen L, Burke WM, Hershman DL, Wright JD (2016). Radiation duration in women with cervical cancer treated with primary chemoradiation: a population-based analysis. Cancer Invest.

[18] Lin JF, Berger JL, Krivak TC (2014). Impact of facility volume on therapy and survival for locally advanced cervical cancer. Gynecol Oncol.

[19] Holschneider CH, Petereit DG, Chu C (2019). Brachytherapy: A critical component of primary radiation therapy for cervical cancer: from the Society of Gynecologic Oncology (SGO) and the American Brachytherapy Society (ABS). Brachytherapy.

[20] McDaniels-Davidson C, Feng CH, Martinez ME (2022). Improved survival in cervical cancer patients receiving care at National Cancer Institute-designated cancer centers. Cancer.

[21] Korenaga TK, Yoshida EJ, Pierson W (2022). Better late than never: brachytherapy is more important than timing in treatment of locally advanced cervical cancer. Gynecol Oncol.

